# Moral Expressions in 280 Characters or Less: An Analysis of Politician Tweets Following the 2016 Brexit Referendum Vote

**DOI:** 10.3389/fdata.2021.699653

**Published:** 2021-07-01

**Authors:** Livia van Vliet

**Affiliations:** Department of Sociology, Amsterdam Institute for Social Science Research, University of Amsterdam, Amsterdam, Netherlands

**Keywords:** twitter, moral foundations dictionary, moral foundations theory (MFT), brexit, political communication, European union, LIWC, linguistic inquiry word count (LIWC)

## Abstract

Ideas about morality are deeply entrenched into political opinions. This article examines the online communication of British parliamentarians from May 2017-December 2019, following the 2016 referendum that resulted in Britain's exit (Brexit) from the European Union. It aims to uncover how British parliamentarians use moral foundations to discuss the Brexit withdrawal agreement on Twitter, using Moral Foundations Theory as a classification basis for their tweets. It is found that the majority of Brexit related tweets contain elements of moral reasoning, especially relating to the foundations of Authority and Loyalty. There are common underlying foundations between parties, but parties express opposing viewpoints within a single foundation. The study provides useful insights into Twitter’s use as an arena for moral argumentation, as well as uncovers the politician’s uses of moral arguments during Brexit agreement negotiations on Twitter. It contributes to the limited body of work focusing on the moral arguments made by politicians through Twitter.

## Introduction

The United Kingdom European Union membership referendum, herein referred to as Brexit, took place on June 23, 2016.51.8% of the voters were in favor of leaving the European Union (EU) and the narrow victory was promised to be implemented. The succeeding government - with Theresa May as the Prime Minister-led the withdrawal process, attempting to negotiate an agreement about the future relationship between the United Kingdom (United Kingdom) and EU (Tzelgov & Dumitrescu, 2018). May’s Prime Minister appointment was marred by political divisions, and she was unable to secure the backing of Parliament on any Brexit deal. Thus, the period of debate about the type of Brexit there should be is of the greatest interest to this article. The negotiations of the departure of the United Kingdom from the EU has been referred to as a ‘divorce process’, wherein agreements needed to be made regarding trade, memberships of certain EU bodies, immigration and so forth. During May’s appointment, certain issues such as immigration and trade could not be agreed-upon (Zappettini, 2019). A second referendum was proposed, as a way to break parliamentary deadlock. Hence, the times following the Brexit referendum were politically tumultuous and deserve further academic scrutiny, especially considering that many of the negotiations involved arguments that were moral in nature.

Political scientists often distinguish moral issues from non-moral (or pragmatic) ones; the latter relies on pragmatic, consequentialist reasoning, whereas the former depends on principles and deontological reasoning (Colombo, 2021). Thus, moral arguments are distinguishable from other arguments as they express moral values - things that ground judgments about what is good or bad, right or wrong, desirable or undesirable (Ryan, 2019). They consist of an expression of political sentiment - positive or negative associations toward moral claims - where the subject matter offers a moral conclusion (Feinberg and Willer, 2013; Feinberg and Willer, 2015). Although not all political arguments can be classed as moral, moral-based arguments hold pervasive power on many different political issues (Feinberg and Willer, 2012) , so much so, that morality is noted to underline much of political decision making (Day et al., 2014; Lipsitz, 2018; Voelkel and Brandt, 2019). Much of the work on political moral decision making is rooted in Moral foundations theory (MFT), which is composed of five foundations that are thought to be responsible for the unique moralities we see across cultures. The foundations have been observed across a variety of cultural contexts, where left and right-wing individuals respond to moral arguments in different ways (e.g., Graham et al., 2009; Strimling et al., 2019). Online social networking websites provide an arena to examine these moral arguments.

Twitter is a micro-blogging social network platform, most often used for news and information dissemination, making it ideal for political research compared to other platforms (del Gobbo et al., 2020). As it is so accessible, Twitter data is often used to research many socio-political issues, such as social movements (e.g., Ince et al., 2017; Ray et al., 2017; Xiong et al., 2019 etc) and political campaigns (see Jungherr, 2016 for a systematic review). Authors in this realm mainly focus on three main areas of interest: election prediction, sentiment analysis of political topics and social analysis of the interaction between politicians and citizens (Korakakis et al., 2017). Tweets - messages of 280 characters or less - are broadcast to large audiences of ‘followers’, or can also be directed to specific members on the platform, in the form of ‘mentions’. Brexit is one of the most prominent and important political events in the last decade -over 135 million tweets containing ‘Brexit’ were made in just a 3 month period: between Dec 2019-Feb2020 (del Gobbo et al., 2020). Hence, Twitter serves as an ideal research point to examine the messages from politicians regarding the Brexit agreement negotiations.

This article aims to study the moral arguments used during the campaign for the Brexit referendum in the Twitter dialogue of politicians. It looks at moral arguments specifically made by political actors - in this case, members of the British parliament, as moral arguments endorsed by political actors are more persuasive than informative arguments from non-political actors (Tzelgov and Dumitrescu, 2018). Moreover, moral frames may explain the high levels of polarization over the negotiations (Feinberg and Willer, 2012; Maher et al., 2018). The main question is; how do British parliamentarians use moral foundations to discuss the Brexit withdrawal agreement on Twitter? The aim is to provide a deeper empirical exploration of tweets made by politicians on Twitter during the UK's withdrawal negotiations from the EU. Initially, tweets are examined for whether or not they contain moral arguments, as the sound bite-style affordances of the platform may not be appropriate for moral argumentation. We are therefore also able to learn more about Twitter communication, especially the frequency in which moral foundations are used by parliamentarians.

### Related Work

#### The Importance of Brexit on Twitter

The Brexit debate has caused rifts between parties, with the parties not being able to agree on the terms for leaving the EU. For instance, Labor was generally more in favor of a friendly deal with the EU, whereas the conservatives were more inclined to want greater (economic)independence (Hobolt, 2018). This political infighting leads to the suggestion that there is a clash of worldviews, potentially on moral grounds. These disagreements may be seen on Twitter, as the content of politician tweets comprises one important part of public politician communication. While one may not immediately expect moral arguments on Twitter (especially due to the restriction of 280 characters), Brexit arguments on other media are largely made on moral grounds (Smith, 2019). Hence, the brief nature of tweets lend themselves to ‘straight to the point’ content, yet it is unclear if moral arguments are pervasive due to the brevity.

Moral and pragmatic (especially economic) arguments surrounded the Brexit referendum vote. For example, the ‘vote remain’ side often argued negative economic consequences if the United Kingdom were to leave the EU, which are usually regarded as more pragmatic arguments (Sampson, 2017). On the other hand, the ‘vote leave’ campaign largely relied on moral arguments to secure the win for the referendum, such as the idea that more money could be given to the national healthcare system instead of going to the EU (Tzelgov and Dumitrescu, 2018; Smith, 2019). It is unclear if these arguments are expressed on Twitter, as while analyses of Brexit data often consists of millions of Tweets (e.g., Khatua and Khatua, 2016; Grčar et al., 2017 etc), few point out specific tweets with clear moral arguments.

Although there have been a number of studies that look at the Brexit debate on social media (e.g., Agarwal et al., 2018; Khatua and Khatua, 2016; Hänska and Bauchowitz, 2017; Grčar et al., 2017; Llewellyn et al., 2019; Lansdall-Welfare et al., 2017; Hürlimann et al., 2016; Usher et al., 2019; del Gobbo et al., 2020), there are few which focus on political sentiments surrounding Brexit on Twitter, let alone moral foundations (Hürlimann et al., 2016; Lansdall-Welfare et al., 2017). Generally, sentiment toward Brexit is inferred from hashtags used, such as #voteleave for positive sentiment toward Brexit (or conversely, negative sentiment toward the EU) or #voteremain for the opposite (Khatua and Khatua, 2016; Usher et al., 2019). On the contrary, other sources such as parliamentary debates may provide thick descriptions of parliamentary discussions, but Twitter is another medium that these discussions can play out in the public eye. Moreover, due to its informal and brief nature, tweets may garner more public attention than the discussions in parliament. Thus, political communication on Twitter is a relatively understudied but important area of research for polarizing and moralizing topics.

Overall the leave campaign used a complex entanglement of moral foundations, especially in the key leaving arguments of healthcare and immigration (Smith, 2019). Following the vote to leave the EU, it is important to adhere to these moral reasonings, as they were the promises made by the vote leave campaigners. In doing so, they can maintain faith in government (Anderson et al., 2020), discourage civil conflict (Outhwaite, 2017) and set the ground for what is wanted from the EU withdrawal agreement, especially regarding money, citizens’ rights and the like. Hence, the period following the referendum is when this moral reasoning can be translated to more concrete ideals set in future legislation. We can better understand these in the way of Moral Foundations Theory (Haidt, 2012).

### Moral Foundations Theory and Political Ideology on Twitter

Moral reasoning underlies political ideologies, and differences in moral judgments can have significant implications for political discourse and relations (Haidt, 2012). The five foundations are:

• Care/harm: focused on caring for the vulnerable, and protecting others from harm.
• Fairness/cheating: the importance placed on equal treatment for all.
• Loyalty/betrayal: the importance of loyalty toward ones in-group.
• Authority/subversion: regards the respect for authority and community rules.
• Sanctity/degradation: mainly concerned with protecting spiritual/religious purity.

Individual sensitivities to the five moral foundations are correlated with political ideologies (Graham et al., 2009). There is growing evidence that left and right wing supporters show preference for different moral foundations to inform their political views (Graham et al., 2009; Haidt, 2012; Graham et al., 2013). When considering the use of moral arguments, it is famously postulated that left-leaning individuals rely more on foundations of care and fairness, whereas right-leaning individuals rely more on loyalty, authority and sanctity (Graham et al., 2009). Other research has found that right-leaning people use arguments related to authority and sanctity (Frimer, 2020). Interestingly, while the values may appeal to left and right-wing individuals differently, violations of these values elicit different reactions. Right-leaning individuals respond more to violations of authority and control, whereas left-leaning individuals react stronger to perceived suffering and unequal treatment (Graham et al., 2013; Haidt et al., 2009). Hence, while the spectrum of moral values may appeal more to right-leaning individuals, violations of authority garnered the strongest reactions.

#### Why MFT Is Important in the Case of the Brexit

In general, the argument for following through with the referendum vote is that it should reflect the will of the people, which can be seen as a moral rather than pragmatic argument. However, ‘Vote Leave’-the official group campaigning for the United Kingdom’s exit from the EU-often attacked the lack of available healthcare by the NHS for British citizens appeals greatly to the foundation of Care, whereas arguments around the issues of immigration-especially concerning those from Islamic nations - were noted as a threat to British Sanctity (Smith, 2019). Interestingly, ‘Vote Leave’ was led by Conservative parliamentarians Boris Johnson and Michael Gove, along with Labor parliamentarian Gisela Stuart. Hence, the campaign had support from both sides of the political spectrum, which is contrary to the research showing that left and right-wing parties tend to place emphasis on different moral arguments (Graham et al., 2009; Haidt, 2012; Graham et al., 2013).

Additionally, voting leave was predicted by political conservatism, social change insecurities, and placing moral importance on personal liberty, relating largely to the foundations of Loyalty and Authority. In contrast, only an adherence to the Care foundation of morality predicted “remain” voting (Harper and Hogue, 2019). This is quite contradictory considering many of the ‘vote leave’ arguments attacking the inability of the NHS to care for British citizens. Overall, Breixt brought to light many different moral arguments which were supported or opposed by parliamentarians on different ends of the ideological spectrum.

There have thus been many studies that examine the Brexit debate, yet none which examine the debate about what kind of Brexit there should be, as in, whether the ‘divorce agreement’ should retain strong ties with the EU, or whether Britain should cut almost all ties—an event known as a ‘no deal Brexit’. As Theresa May’s government came into power following the referendum vote, this is the legislative period which is focused on, as data from two parliamentary periods should not be mixed. This research contributes to the body of knowledge on the presence of moral foundations in parliamentarian tweets, especially in the case of Brexit. It asks; how do British parliamentarians use moral foundations to discuss the Brexit withdrawal agreement on Twitter?

To answer the main question, several aspects are examined, focusing especially on the frequency of moral arguments, the key terms associated with each foundation, and the differences between parties. From the literature, both the null and alternative hypotheses are considered when it comes to the use of moral foundations by parliamentarians:

**H**_**0**_ **:** There will be few tweets that contain moral arguments, due to the limited number of characters available for complex moral expression.

**H**_**1**_ **:** There will be a proportion of tweets that contain clear moral arguments.

Secondly, the literature stating that different ideologies rely on different moral foundations to argue their position on the Brexit agreement is also considered (Graham et al., 2009; Haidt, 2012; Smith, 2019), and further hypothesize:

**H**_**2**_ **:** Left-leaning parties (Labor and Labor Co-op) will focus on arguments centered on Care and Fairness, whereas Conservatives will use a wider variety of moral arguments.

First the methods will be outlined, which involves a rigorous hashtag selection process, followed by the construction of a Brexit-specific dictionary. Then the results are presented in the order of the hypotheses outlined. Finally, the results are discussed in light of the moral arguments found in parliamentarian Brexit tweets.

## Methods

**FIGURE 1:**
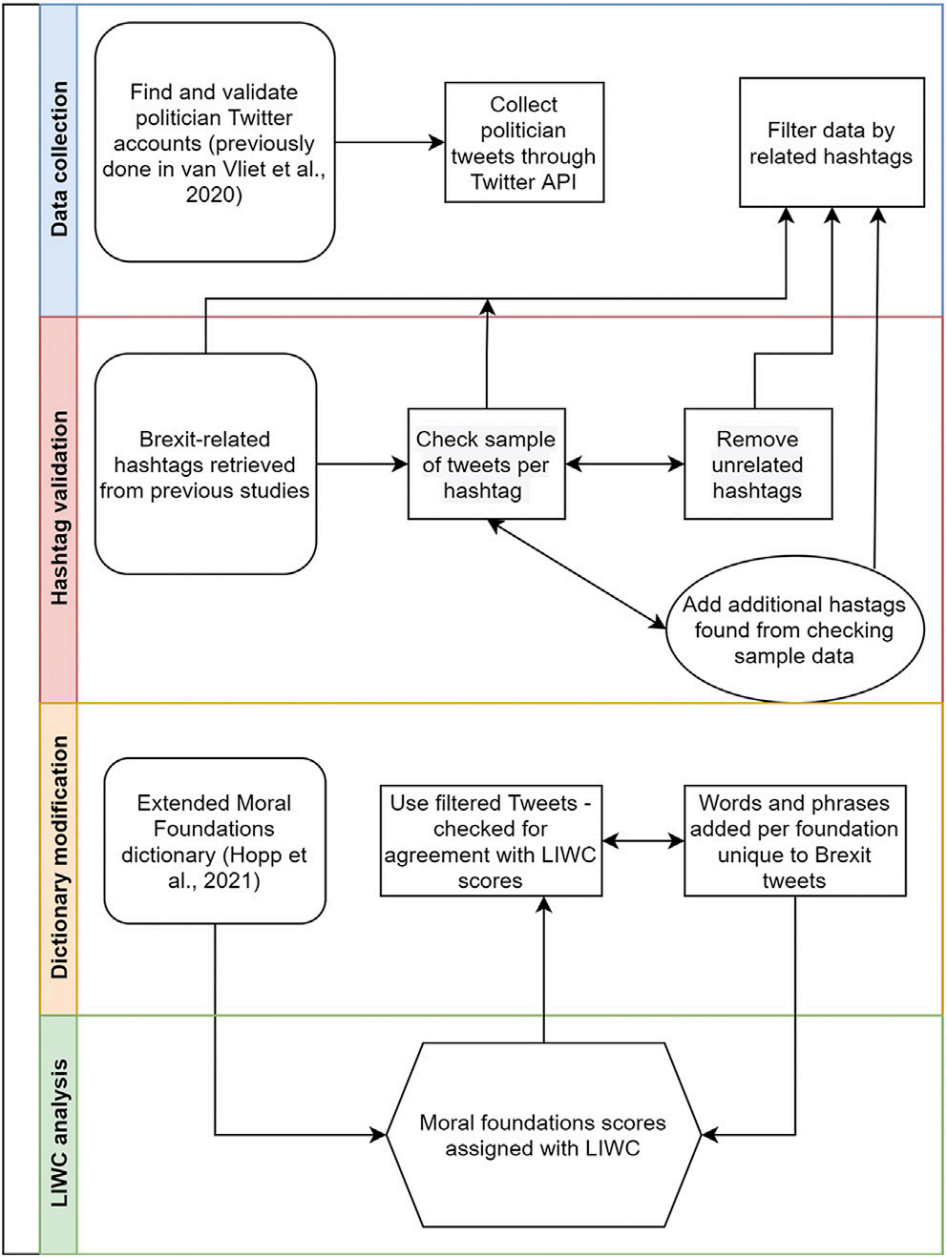
Process diagram showing the iterative methods in hashtag selection and dictionary modification.

The entire stream of tweets from 590 British Parliamentarians was gathered using Twitter’s Streaming API from June 1, 2017 until the election of the new parliament on December 12, 2019 (van Vliet et al., 2020). During this time, there were parliamentary deadlocks on what exactly would happen in the divorce process with the EU. This date was also chosen because it is prior to the entanglement with SARS-CoV2. For the analysis, retweets were removed, as retweets represent moral arguments which may be echoed or endorsed, rather than those which are stated by the parliamentarians themselves. With retweets removed, 30,122 tweets from British parliamentarians regarding Brexit were analyzed. The process model for the methods can be seen in Figure 1.

### Identifying Brexit Tweets

From the database of tweets from 590 incumbent British Parliamentarians, tweets about Brexit were first identified. Hashtags were used to filter the tweets, which were selected through an iterative process. Firstly, tweets were extracted using the Brexit related hashtags from Bastos and Mercea, 2018, as well as related tags from the website Ritetag (Ritetag, 2020), which shows related hashtags to a specific query. The parliamentarian tweets were also searched for any hashtags containing “brexit” and added to the selection list. From this list of hashtags, a sample of 100 tweets was labeled as being relevant to Brexit or not. In the cases where there were less than 100 tweets for that hashtag, all tweets were labeled (*N* = 3,492). During the labeling, more related hashtags were uncovered and also validated for their relevance. From there, only hashtags that had over 100 tweets with 95% of them directly related to Brexit were selected for the analysis. Finally, retweets were also excluded as a main aim of this article is to identify the moral arguments directly made by politicians, rather than those disseminated or endorsed by them. The final list of hashtags used in the analysis are outlined in Table 1. Through this process it was found that politicians generally use hashtags for issue positioning, in line with literature (Hemphill et al., 2013; Enli and Simonsen, 2018; Barberá et al., 2019), and some hashtags that were used by the public regarding brexit (e.g., #moreincommon) were used for a completely unrelated event by politicians. Overall, hashtag selection is extremely important to be able to narrow the analysis down to a specific subject. Broader tags like #cor and #theresamay are not narrow enough with regards to brexit and hence were left out of the analysis.

**TABLE 1:** Hashtags used in the analysis. Retweets are excluded.

Hashtag	All tweets (*N*)	Retweets (*N*)	Mentions (*N*)	Related tweets (%)
Brexit*	37,056	15,078	21,978	100
Getbrexitdone	2,589	1,044	54	100
Stopbrexit	2,143	462	1,652	100
Nodeal*	1,643	554	1,089	100
Exitfrombrexit	671	37	634	100
BackTheBrexitDeal	496	246	250	100
revokearticle50	481	170	311	100
article50	424	173	248	99
StandUp4Brexit	353	266	87	100
Remainer	244	123	119	98
Euref	240	109	125	100
RoadtoBrexit	192	98	94	100
Backstop	137	50	86	99
Hardbrexit	126	44	70	100
Britainbeyondbrexit	109	44	62	100

### Quantifying Moral Foundations

The tool used to label the tweets by their moral foundations is Linguistic Inquiry Word Count (LIWC). LIWC calculates a percentage of words in a corpus that belong to several predefined categories (Tausczik and Pennebaker, 2010). In our case, these categories are the five moral foundations: care, fairness, loyalty, authority and sanctity. The percentage of words per category is calculated over a text where all words are given the same weight, and a score per category is calculated for the text. Past studies on the empirical validity of LIWC have found that it is able to detect meaning from texts, including emotional states, motivations and thinking styles (Chung and Pennebaker, 2018).^1^

Several studies have employed the tool Linguistic Inquiry Word Count (LIWC) for studying moral foundations in highly politicized arenas, such as the ‘Ground Zero Mosque’ (Dehghani et al., 2014), stem cell research (Clifford and Jerit, 2013), entertainment media (Ji and Raney, 2015) and immigration (Grover et al., 2019). It was also used by Harper and Hogue (2019) to study moral intuitions regarding Brexit vote intentions. In the domain of political tweets, LIWC has thus far been applied to various political issues (Day et al., 2014; Johnson and Goldwasser, 2018; Alizadeh et al., 2019; Grover et al., 2019), but there are no studies yet which focus on the moral foundations of tweets of politicians surrounding Brexit.

The moral foundations dictionary is a pre-built set of words that enables LIWC to label texts by their moral underpinnings, and assigns a numerical score to the tweet based on the intensity of moral undertones the higher the numerical assignment, the higher the intensity of that foundation. This results in a numerical figure per category, indicating the moral intensity of that foundation within the tweet. For example, a tweet may score 8 in Authority and three in Loyalty, indicating a more intense expression of Authority. Thus, a tweet can be labeled as having elements of care, fairness and loyalty with equal intensity for each, or higher intensity for one foundation over others.

LIWC is used primarily because 1) it has foundations in social science research and has been used in similar research contexts (e.g., Dehghani et al., 2014; Grover et al., 2019) and 2) the moral foundations dictionary (MFD) built for LIWC is theoretically refined and has the most suitable existing lexicon for testing our hypotheses (Hopp et al., 2021; Frimer et al., 2019; Graham et al., 2009). The dictionaries contain word stems that are designed to deal with singular/plural forms of words, and also include lemmas for several terms. Hence, LIWC is known for its methodological and theoretical consistency in researching moral foundations in tweets. As noted, the dictionary used with LIWC provides multi-label output, meaning that more than one foundation can be detected per tweet.

### Validating the Dictionary

In the analysis an updated version of the MFD is used, the extended Moral Foundations Dictionary (eMFD) (Hopp et al., 2021). A sample of tweets (*N* = 300) was taken and manually labeled based on their foundations. Out of the box, the eMFD agreed with manual labeling 66% of the time, across moral and non-moral Brexit tweets. To increase labeling accuracy, the eMFD was amended to make it more Brexit-specific. During labeling, specific Brexit-related words and phrases were noted, such as issues of immigration usually being related to sanctity, and most tweets mentioning Theresa May or Boris Johnson were to do with either questioning or praizing them as an authority. The mislabeled tweets were also examined, and the eMFD was further amended based on these errors. For instance, only two words were removed: ‘faith’ and ‘lords’ from the terms for sanctity. Prior to removal of the words, all tweets containing ‘lord’ (*N* = 197) and ‘faith’ (*N* = 85) were checked, and found that they were not at all related to religion, but rather about having faith in people, or the titles for people, or referring to the house of lords. Terms surrounding immigration, Islam and Turkey were added, as in the case of the Brexit, they are noted to be largely related to sanctity (Smith, 2019).^2^ It was crucial to add these terms as well as remove ‘lord’ and ‘faith’, for more accurate labeling of the data. Without the removal, at least 170 tweets would be mislabeled as relating to sanctity, resulting in an erroneous overrepresentation of this foundation.

To ensure the dictionary was not just modified to suit the sample tweets, two trained coders manually labeled another random sample (*N* = 200). The coders followed the same coding guidelines from Hoover et al. (2020), as well as some extra notes on Brexit-specific issues, such as those on immigration and healthcare. The coding guidelines can be found in Supplementary Information Section 1.1. For all foundations, the coders were in agreement for over 85% of cases. Krippendorf’s alpha (α) produced high scores for Care (α = 0.81), Authority (α = 0.72) and Non-moral (α = 0.86) labels, however the results were lower for the less-used foundations, such as Loyalty (α = 0.59), Fairness (α = 0.64) and Sanctity (α = 0.39), despite having a high percentage of agreement between coders.

The coders agreed with over 75–81% of the labels from the Brexit-adapted dictionary agreed with manual labeling (whereas the initial sample resulted in 87% agreement). This shows that the adaptation of the dictionary to Brexit-specific terms results in an overall improvement in accuracy, and that we did not only create the dictionary based on the sample data. Finally, we note that human agreement with moral labels is not perfect, and agreement can range from 66–95% depending on the study and method of measuring agreement (Weber et al., 2018). Therefore we find the level of 75% agreement acceptable. In the Supplementary Information (Table 1) are examples of tweet labels assigned by LIWC.

## Results

### The Frequency of Moral Arguments

To test the first hypothesis - that there will be a proportion of tweets that contain moral arguments we looked at the proportion of tweets that were assigned a score on any moral value with LIWC. From the tweets extracted (*N* = 30,122), 65% (*N* = 19,760) contained some element of a moral argument. We can confirm H_1_ there are a proportion of Tweets that contain moral arguments however we are not fully able to confirm a lack thereof, as LIWC is only able to test the presence of certain words, and not tweets that may be laden with moral judgements without explicitly stating them. Figure 1 shows the moral labeling of the tweets, where 38% (*N* = 11,374) of tweets contained some element of Authority, which is more than those that were labeled as having no moral underpinning (*N* = 10,362). Thus, with regards to the first hypotheses, we see that the majority of tweets do contain moral underpinnings. Authority was the most frequently used foundation, followed by Loyalty (31%) and Care (17%).

As LIWC is a frequency counter that produces multi-label output, we further test if there are correlations between foundations, to determine if two foundations are often used together in one tweet. Due to assumptions of normality being violated, Spearman’s Rho was used to test the correlation between two foundations (*N* = 30,122). It was found that there are several negligible but significant relationships between several of the foundations. For instance, Fairness correlates positively with Care (*r*_*s*_ = 0.029, *p* = 0.000), Authority (*r*_*s*_ = 0.042, *p* = 0.000) and Sanctity (*r*_*s*_ = 0.019, *p* = 0.000). This means that arguments rooted in Fairness are likely to also contain elements of Care, Authority and Sanctity. For example, one parliamentarian tweeted:

“The Government’s plan for #Brexit will make it harder to bring international drug gangs to justice. By losing the European Arrest Warrant and information sharing arrangements, these criminals will be much harder to catch. #ExitFromBrexit”.

Within this tweet there is the argument of Fairness (justice against international drug gangs), Care (caring for the safety of the population by reducing criminality), Authority (the European Arrest Warrant sharing agreement) and Sanctity (protecting the purity of the population).

Loyalty on the other hand only correlates very slightly positively with Care (*r*_*s*_ = 0.015, *p* = 0.010). Thus, arguments rooted in loyalty also may contain elements of Care. For example, another parliamentarian tweeted:

“Half of doctors from other EU countries considering leaving United Kingdom, a fifth already made plans, 89% fewer EU nurses coming #Brexit”.

Within this tweet we see the entanglement of Care and Loyalty foundations, where healthcare workers are considering leaving the United Kingdom, and hence showing loyalty to the EU and reducing the healthcare capacity in the United Kingdom. Generally, the correlations were very weak and can be found in Supplementary Table S2 in the supplementary information.

#### Moral Arguments Over Time

In order to better understand the data before looking at the moral arguments within, the distribution of the most frequent hashtags was examined (see Table 1). It was found that in line with previous studies (e.g., Khatua and Khatua, 2016), some of the hashtags used by parliamentarians already contain an element of moral judgment. For example, #BackTheBrexitDeal, #getbrexitdone and #StandUp4Brexit are in support of the current Authority to go through with Brexit and the proposed agreements, and are sometimes mixed with tweets about Loyalty to Britain over the EU. On the other hand, #stopbrexit and #revokearticle50 are in direct opposition of it and are used in support of the Authority of the European Union as well as Loyalty to the EU. We see from Table 1 that #brexit was the most commonly used, followed by #stopbrexit and #nodeal. It should be noted that the hashtags are grouped by their word stem, so #brexit also contains the tags #brexitchaos #brexitshambles #brexitdeal and so forth. These hashtags were often used alongside one another too.

**FIGURE 2:**
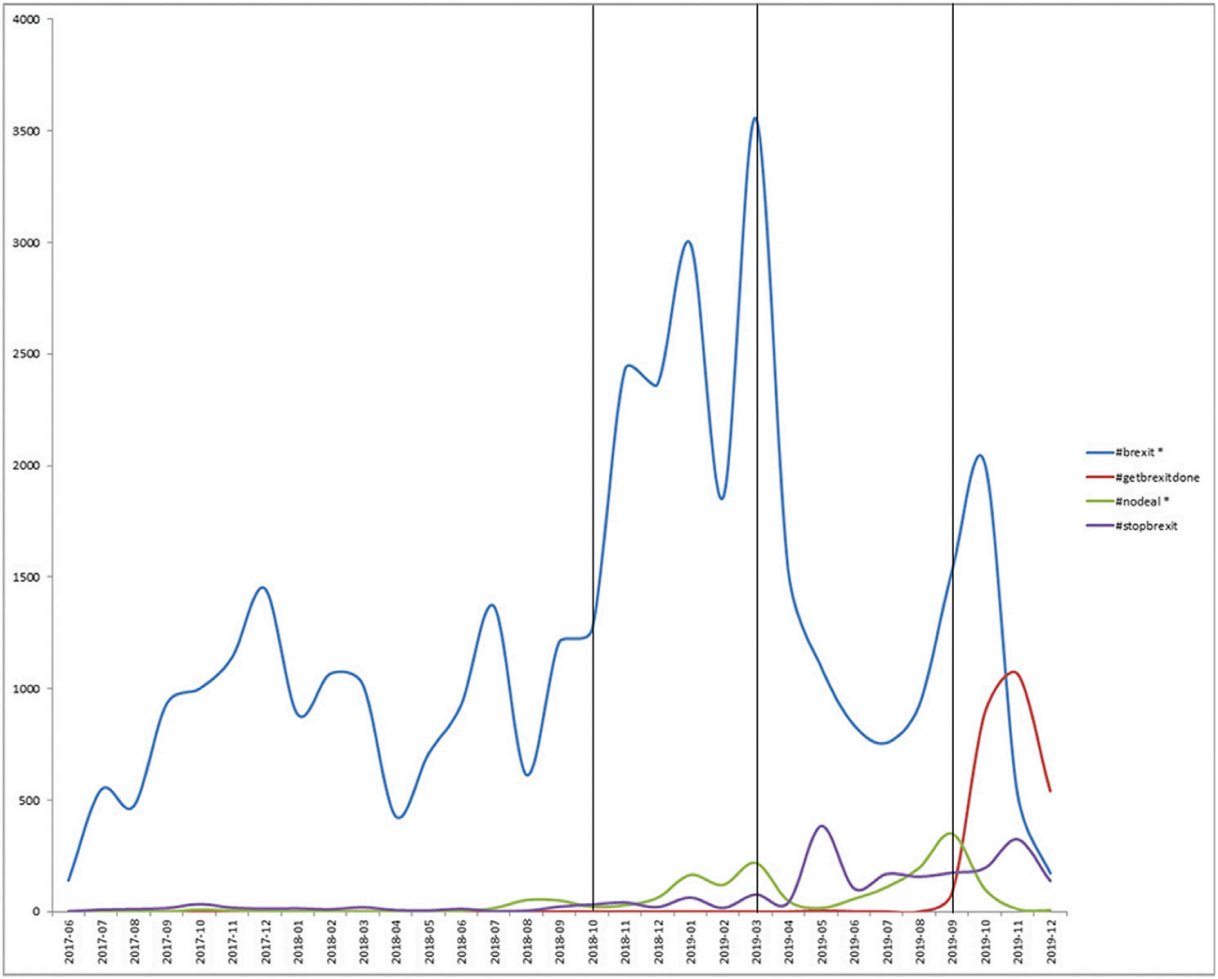
Line graph showing the hashtag frequency over time. Hashtags denoted by * also include derivatives of that hashtag as a word stem.

To first see if the moral arguments differ over time, we look at the hashtag distribution over time. We see from Figure 2 that #brexit was clearly the most used hashtag, and others were only used for certain periods. There was a large general increase in Brexit related tweets from November 2018 april 2019, with a large peak of #brexit hashtag activity in March 2019. The activity then dropped significantly until October 2019, where it rose again, alongside the #getbrexitdone hashtag, which was largely unused until September 2019. This largely coincides with campaigning times, with elections being held on December 12 2019, since #getbrexitdone was a slogan for Boris Johnson’s campaign for the Conservatives.

**FIGURE 3:**
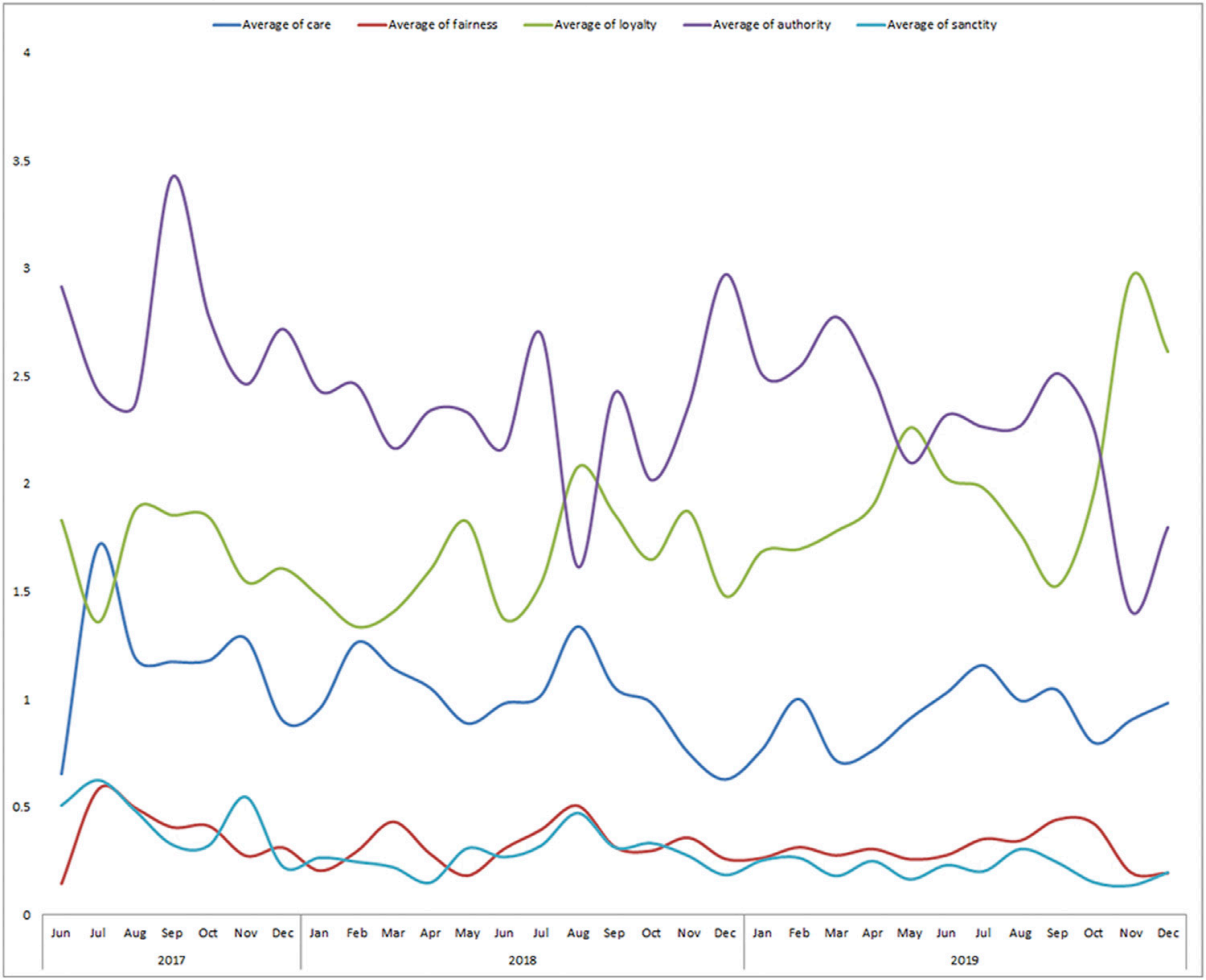
Line graph showing the average moral intensity of Brexit tweets over time. The foundations of authority and loyalty were most strongly used. This shows that moral arguments overall are sustained over time.

Once the tweets were labelled, we checked to see if there were differences in the intensity of moral arguments over time, depending on what might hold the public interest. From Figure 3, we see that the average intensity of Fairness and Sanctity generally remains the same. However, there are fluctuations over time in the average intensity of Authority, Loyalty and Care. On average, the arguments of Authority and Loyalty were used the most intensely over time. Overall, parliamentarians appealed most often to the foundation of Authority with regards to Brexit. This makes sense, due to many arguments questioning and challenging authority, such as the competence of Prime Minister Theresa May in creating a deal the cabinet could agree with, or to support Boris Johnson’s new proposed deal.

### Key Terms Associated With Each Foundation

**FIGURE 4:**
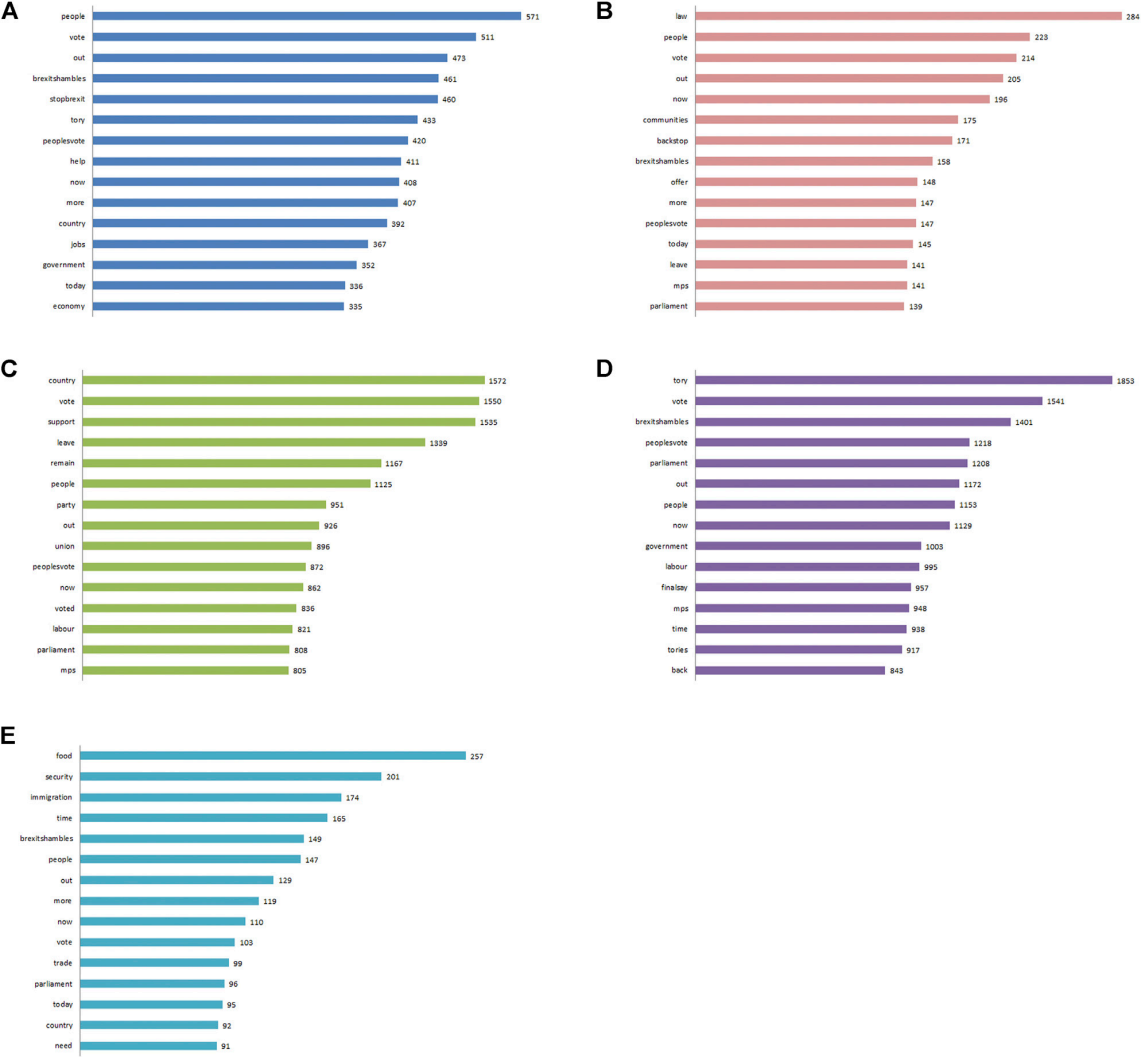
Word clouds and frequencies per foundation - Care **(A)**, Fairness **(B)**, Loyalty **(C)**, Authority **(D)** and Sanctity **(E)**. Words are sized by their frequency and colored randomly. Frequency bars for words containing 3 characters or more are shown on the right.

Previous literature suggests that arguments rooted in Care will primarily involve the NHS, whereas those centered on Loyalty and Sanctity will be more related to immigration and the backstop (Smith, 2019). Figure 4 visualizes word frequencies per foundation, with the removal of stopwords, including ‘Brexit’, ‘EU’ and ‘deal’. From this figure we see that tweets labeled with Care discusses ‘people’, ‘United Kingdom’ and ‘jobs’, although these words are generally outnumbered by ‘StopBrexit’, and ‘PeoplesVote’. We also see that tweets labeled with Loyalty mainly discuss ‘United Kingdom’, ‘support’, ‘vote’ and ‘parliament’, whereas tweets categorized with Sanctity tend to discuss ‘immigration’, ‘food’ and ‘people’. These findings somewhat are in line with previous literature, with arguments relating to Sanctity discussing immigration and the backstop (Smith, 2019).

After removal of stopwords, the 10 most frequent words per moral foundation that were not commonly shared with the other foundations were extracted (i.e., the word did not appear in the top 50 most frequently used words in the other foundations), and calculated the log-likelihood (LL) value to indicate overuse or underuse respectively, in one foundation relative to tweets that are not labeled in that foundation (Boukes and Trilling, 2017; Arlt et al., 2019). In other words, the LL value shows how frequently a word appears in one group of tweets over another (i.e., belonging to one moral foundation over others). If a word occurs more or less frequently than expected by chance in one of the groups of tweets, the higher the LL value is. We further calculated the probability based on the chi distribution to determine if the frequency difference was statistically significant. The results can be found in Supplementary Table S3 in the Supplementary Information.

It was found that ‘help’ (*p* = 0.000), ‘jobs’ (*p* = 0.002), ‘fight’ (*p* = 0.000) and ‘damage’ (*p* = 0.000) were significantly more likely to appear in tweets categorized with Care over other foundations. Interestingly, the NHS was not significantly mentioned more in tweets regarding Care. Words such as ‘law’, ‘community’, ‘offer’, ‘blame’, ‘fair’ and ‘honest’ were all significantly more likely to appear in the Fairness foundation than others (*p* = 0.000 for all). For the Loyalty foundation, the words ‘union’, ‘customs’, ‘local’ and ‘together’ appeared significantly more in those tweets (*p* = 0.000 for all). For Authority, the most significant words were ‘finalsay’, ‘tories’, ‘theresa’, ‘prime’ and ‘boris’ (*p* = 0.000 for all). Finally the words ‘food’, ‘security’, ‘immigration’, ‘clean’, ‘bill’ and ‘money’ were significantly more likely to appear in tweets labeled with Sanctity (*p* = 0.000).

Although words may appear more frequently in one foundation over others, when compared with the rest of the text (and not directly to another foundation), only around 5 words were said significantly more in each foundation over others (See Supplementary Table S3). Moreover, some words were used significantly less in the labeled foundation when compared to the rest of the tweets. Lastly, it should be noted that due to LIWC being dictionary-based, certain words were consistently categorized as belonging to a certain foundation, and thus did not appear at all in the rest of the text as they were exclusively assigned to a certain foundation. This happened commonly with the Fairness foundation, with words such as law, community and fair.

### Differences in the Use of Moral Foundations per Party

**FIGURE 5:**
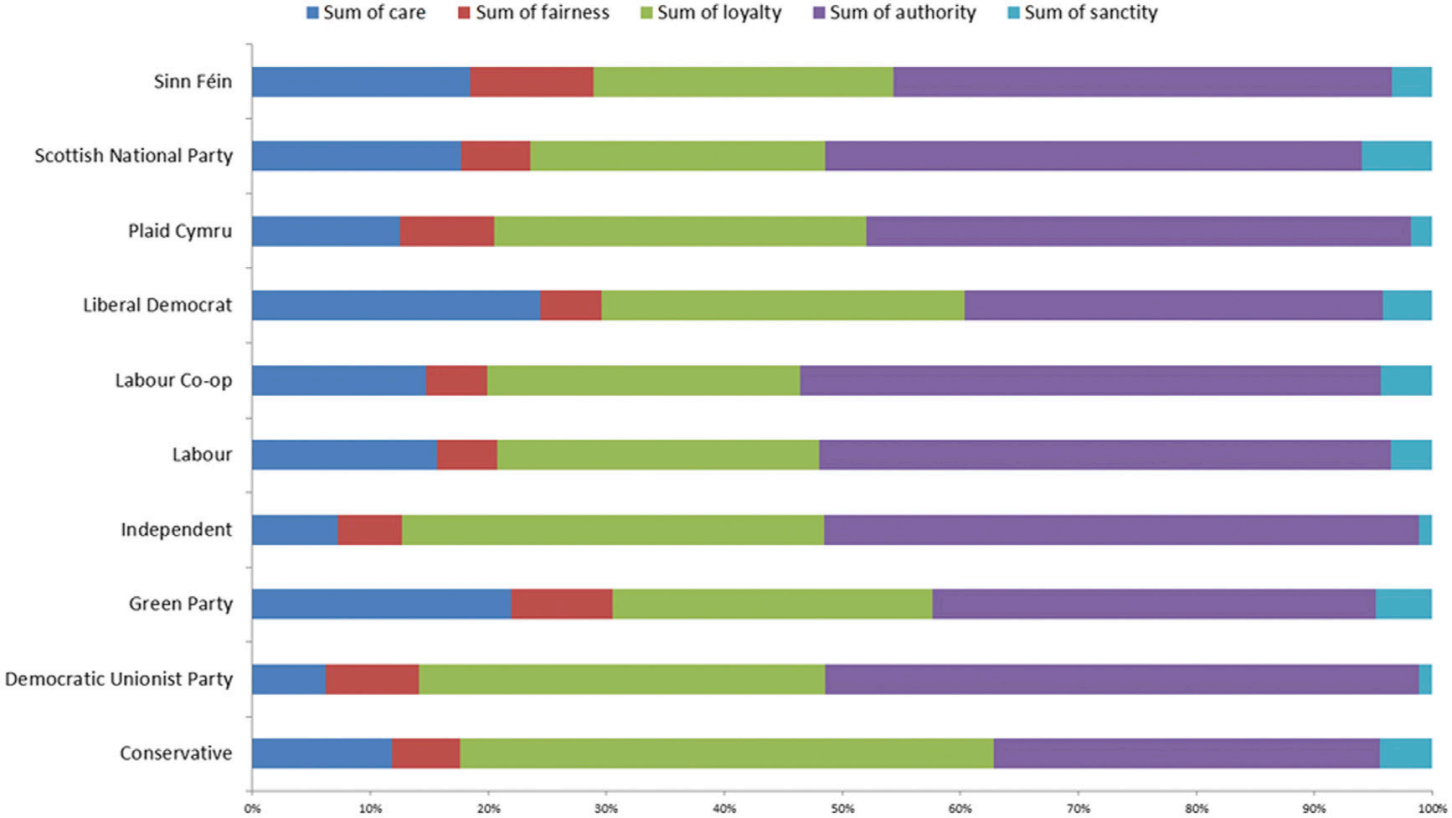
Stacked bar graph showing the proportion of moral intensity for each moral foundation, per party. We see that most parties generally used arguments of Authority, Loyalty and Care.

It was hypothesized that Labor would focus on arguments centered on Care and Fairness, whereas the Conservatives will use a wider variety of moral foundations in their arguments (Graham et al., 2009; Haidt, 2012). Figure 5 shows the proportion of moral foundations per party. Most parties use arguments of Authority, Loyalty and Care. However there is a marked difference between the proportion of tweets labeled with Care between the Labor party and the Conservative party, where the bulk of moral tweets by the Conservative party focused on Loyalty and Authority.

**FIGURE 6:**
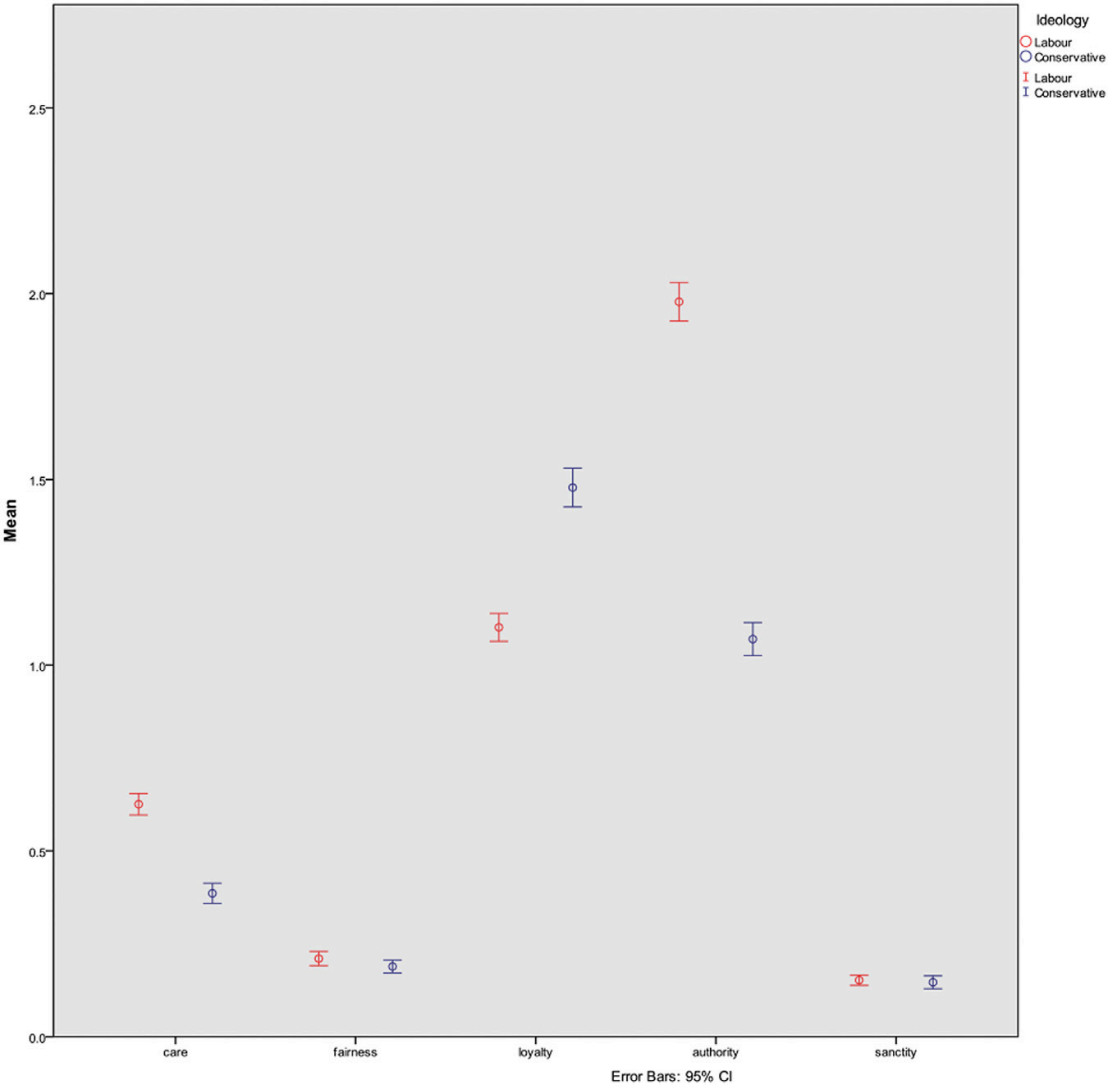
Mean differences with error bars for moral intensity per foundation between Labor and Conservatives. There are significant differences in the use of Care, Loyalty and Authority.

To further test H_2_, the two largest parties were examined Labor and the Conservatives who had the largest volume of Brexit-related tweets: 39 and 27% of all Brexit related tweets were issued by Labor and Conservatives, respectively. A one-way ANOVA was conducted to compare the means of moral intensities between the two parties, to determine if there was a statistically significant difference in how intensely each party expresses certain moral foundations. We found that there were significant differences between Care (x̅_Labor_ = 0.62, x̅_Conservative_ = 0.38, *p* = 0.000), Loyalty (x̅_Labor_ = 1.10, x̅_Conservative_ = 1.48, *p* = 0.000) and Authority (x̅_Labor_ = 1.198, x̅_Conservative_ = 1.07, *p* = 0.000). These mean differences can be seen in Figure 6. Thus, those in the Labor party appeal more intensely to the foundations of Care and Authority than Conservatives, whereas Conservatives appeal more to the foundation of Loyalty. There were no significant differences in the foundations of Fairness and Sanctity.

The differences in intensity however, do not mean that each party discusses each foundation in a similar way. Taking a random sample of tweets per party (*N* = 100 per party), it was found that the parties approached the foundations in differently. For instance, as we see from Figure 5, most parties do appeal to the foundation of Authority, yet discuss them in different ways Labor often criticized the competence of the cabinet to be able to go through with creating an agreeable deal, whereas the Conservatives discuss Authority in more positive terms, such as respecting and supporting the cabinet responsible for implementing a Brexit deal. Thus, the Labor party undermining authority could result in a response from (or be a response to) the Conservatives in support of the Brexit deal and the responsible authority. The same was found for arguments rooted in Loyalty: the Conservatives spoke of loyalty toward the United Kingdom and its citizens, whereas Labor emphasized loyalty toward the European Union.

## Discussion and Conclusion

There were several moral frames used to discuss Brexit by British parliamentarians on Twitter. The majority of tweets (65%) were rooted in at least one moral argument. The rather frequent use of moral frames may explain the high levels of polarization over the issue (Feinberg and Willer, 2012; Maher et al., 2018). Indeed, during the manual labeling and validation of the tweets, there were a surprising amount of negative tweets between parties especially those attacking the opposition often calling into question the competence of other parties as well as the current leadership. Therefore there were clear contentions between parties.

Moreover, hashtag and tweet validation was a critical step in the process not only for ensuring relevant data was analyzed, but also for better understanding how politicians use Twitter. For instance, with the #brexit tag, since the study focused on politician only data, there were no irrelevant tweets or people piggybacking on the hashtag, which is common when looking at unfiltered Twitter data. Thus, in line with present research, politicians generally use hashtags to strictly demarcate specific issues (Hemphill et al., 2013; Enli and Simonsen, 2018; Barberá et al., 2019). It was further found that the hashtags used by politicians also differed from those used by the public (e.g., Bastos and Mercea, 2018). For instance, the hashtag #strongertogether was used for a totally different event that was not Brexit related.

Most surprisingly, Labor and Conservatives both appealed to similar foundations, especially Authority and Loyalty, but expressed arguments to these foundations in different ways. For instance, concerning Authority, Labor would call into question the authority and competence of Theresa May to get support for her proposed Brexit agreements. The Conservatives on the other hand, appealed to the authority of the cabinet and called for support for the proposed agreements. The expression of Loyalty also differed between the parties, where Labor expressed loyalty to the European Union, but Conservatives expressed loyalty toward the United Kingdom and the British people. Therefore, the difference in how parties use each foundation is a topic for further research.

### Limitations

Using a pre-built labeling program such as LIWC is not without its limitations. From a technical standpoint, it is unclear how LIWC deals with things like typos and word stems. From a theoretical standpoint, moral foundations are ambiguous and mixed, and in this case it is unclear to the extent which tweets were supporting or protesting certain aspects of Brexit. Thus, the virtue and vice judgements were removed, as virtue terms may differ depending on which issues a politician was in support of (e.g., loyalty to the United Kingdom or loyalty to the EU). Omission of the moral valence of the foundations therefore limits the study only to which moral arguments were used, but not which types of virtues were favored by each side.

Moreover, like human coders, LIWC cannot perfectly label tweets. The dictionary-based approach does not take words in their context and can therefore mislabel foundations simply based on the presence of a certain word. This is shown through using the most frequent terms to analyze the differences in word frequency between the foundations - with some words it essentially resulted in reverse-engineering the dictionary. That said, it worked surprisingly well after amending the dictionary, bringing coder agreement with the labeling up to 81%. However, multi-label output can be difficult for drawing succinct conclusions, and thus we can only discuss the intensity of a certain foundation within a tweet, rather than the core idea of the argument behind it. Other open source projects could be tested and compared with LIWC for better labeling of the data.

Another limitation is the selection of data. Although it was carefully attempted to look at a wide variety of hashtags tied to Brexit, Brexit issues and the referendum, it is not sure that all Brexit-related tweets are included. Moreover, members of European Parliament are not included in the analysis, and may play a key role in communicating and disseminating moral arguments to their fellow politicians and constituents.

## Conclusion

This study has examined the Brexit debate between British parliamentarians on Twitter. The study focused on the question; how do British parliamentarians use moral foundations to discuss the Brexit withdrawal agreement on Twitter? Most tweets analyzed were using the hashtag #brexit (or a derivative of it), followed by the ideologically laden hashtags #getbrexitdone and #stopbrexit. The frequency of use of these hashtags changed over time, where hashtags like #getbrexitdone started gaining popularity in the last 3 months of 2019, and was closely associated with Boris Johnson’s campaign for the upcoming elections. The results could confirm H_1_, as a large proportion of tweets contained clear moral arguments were found. In fact, the majority of tweets about Brexit contained moral underpinnings. The most frequently labeled foundation was Authority, followed by Loyalty. Authority was also the most intensely used, indicating that Authority was the prominent foundation for most of the moral tweets.

When looking at the content of these arguments, the literature postulated that arguments rooted in Care will primarily involve the NHS, whereas those centered on Loyalty and Sanctity will be more related to immigration and the backstop. Indeed, we did find that arguments related to Care did mention the NHS, although this was not statistically significant. Instead, Care was significantly related to ‘help’, ‘jobs’, ‘damage’ and ‘fighting’. On the other hand, Sanctity was related to ‘immigration’ and ‘security’, and Loyalty was more about ‘customs’ and (the European) union, with those words being statistically more likely to appear in tweets categorized with those foundations. Conversely, in the case of immigration, this significant difference makes sense due to ‘immigration’ being one of the key words added in the dictionary for the Sanctity foundation.

It was also hypothesized that left-leaning parties will focus on arguments centered on Care and Fairness, whereas Conservatives would use a wider variety. Proportionally, both Labor and the Conservatives tweeted most intensely with arguments rooted in Loyalty and Authority. One-way ANOVA found that indeed Labor focused significantly more on arguments of Care and Authority, but interestingly Conservatives focused significantly more on arguments of Loyalty. Moreover, although both parties used arguments of Authority intensely, the expression of the foundation was different in each party. Labor used it to question the current cabinet, whereas the Conservatives used it in support of it. Similarly, Loyalty was expressed in different ways between the parties, where Labor indicated loyalty toward the European Union, and the Conservatives spoke of loyalty to the British people. Thus, the same foundation was used by parties in different ways.

All in all, the study finds that there are moral arguments from parliament in Brexit-related tweets. Different moral arguments are used by the parliamentarians and the intensity of these arguments differs between Labor and Conservatives. Arguments may appeal to the same foundations yet be used in very different ways, depending on the underlying ideology. This work contributes to the growing body of knowledge over the use of moral arguments by politicians, especially in public online settings.

## Data Availability

The datasets presented in this study can be found in online repositories. The names of the repository/repositories and accession number(s) can be found below: https://doi.org/10.6084/m9.figshare.14465445.

## Footnotes

1 However, the virtue and vice labels were removed, as what is virtuous depends on which issues that a politician supports (e.g. loyalty to British citizens, or loyalty to the EU).

2 The link to the full Brexit-specific dictionary can be found in the Supplementary Information section 1.1.
