# Supplementary material for: Moral Expressions in 280 Characters or Less: An Analysis of Politician Tweets Following the 2016 Brexit Referendum Vote
Source: Front Big Data. 2021 Jul 1;4:699653. doi: 10.3389/fdata.2021.699653 (PMC8281012; doi:10.3389/fdata.2021.699653)
Supplement: Supplementary file 1 [file Table1.DOCX]

Supplementary Material

# Supplementary Data

**1.1 Moral Foundations Dictionary [Brexit Specific]** – can be found at the link https://figshare.com/s/a4a83fde5ebfe00c9f58 with the doi: 10.6084/m9.figshare.14465445

**1.2 Coder guidelines: Brexit specific**

Coders were instructed to follow the annotator instructions from Hoover et al. (2020), as well as the following Brexit-specific guidelines:

**Care/harm -** Often to do with healthcare and the NHS, but can also be to do with the welfare state

**Fairness/cheating –** Which deal is more balanced, is one side favored over the other

**Loyalty/betrayal –** Can be to do with loyalty to the UK / British people OR loyalty to the EU and its traditions

**Authority/subversion –** This also has to do with questioning/praising the authority of Theresa May (who was in power at the time of these tweets), or proposing a new, more competent authority (e.g. Borris Johnson)

**Sanctity/degradation –** The "threat" of Islam to the Christian values of the UK, as well as anything to do with keeping Britain "pure", including keeping out immigrants

# Supplementary Tables

**Table 1: Tweet examples with LIWC classification and the agreement with manual classification**

| **Tweet** | **Foundation scores** | **Party** | **Manual classification** |
| --- | --- | --- | --- |
| #Brexit was supposed to be about UK Parliamentary sovereignty & taking back control. Boris Johnson makes clear what has always been the case, for Tory hard Brexiteers its about *them* taking control. We can't allow that to happen #StopTheCoup https://t.co/T2R9diRo66 | Authority (13.96) | Labour | Authority, Loyalty |
| @ColinBaldy Ordinary MPs like me don’t get the figures. Some evidence of some leaving over #Brexit in my own area, but that’s the very worst thing to do at this vital moment & when I speak to them I can usually persuade them to stay. | None | Labour | Loyalty |
| Tory #contempt for @HouseofCommons knows no bounds - regularly ignore opposition motions, and now PM’s short A50 extension request effectively contradicts *her own motion* agreed last Thurs, saying if no deal had been agreed by today then longer delay wd be needed #BrexitShambles https://t.co/Fkke7S00c2 | Authority (4.34) | Scottish National Party | Authority |
| .@accessjames #CorbynsLabour conned ppl on scrapping tuition fee debt & they're conning ppl again. Let's be clear #Labour support #Brexit | Care (4.76)  Loyalty (4.76) | Conservative | Loyalty, Care |
| Scottish Tories have long accused SNP politicians of using #brexit to talk up Scottish independence, but the number of times Tory Ministers have talked about the linkage between the two in the #BrexitDebate it is almost becoming a self fulfilling prophecy | Loyalty (2.44)  Authority (4.88) | Scottish National Party | Loyalty, Authority |
| The threat of a No Deal Brexit looms ever larger and it is clear that, whether we get a Brexit deal or No Deal, Brexit will damage to our communities and economy. If you @JDjanogly agree that harm will be caused by #Brexit , back a #finalsayonthedeal https://t.co/Um5abZmoE1 | Care (6.0)  Fairness (2.0) | Liberal Democrat | Care, Fiarness |
| Raab can’t answer Peter Bone’s #pmq on whether we’ll leave the EU on October 30th with a simple “yes”. Telling. #brexitshambles #FinalSay #PeoplesVote #PMQs | Loyalty (4.17)  Authority (4.17) | Labour | Loyalty, Authority |
| #awks #brexithaos | None | Labour Co-op | None |
| So tomorrow Britain will have a new PM - but with 100 days to go until #Brexit it’s the red lines that need to change, not the faces - the Withdrawal Agreement will not be reopened!! https://t.co/Kwq8be09b5 | Authority (2.63) | Sinn Féin | Authority |
| @i_mac123 Eliminates the need for transition and backstop and allows for a cleaner Brexit. | Sanctity (6.67) | Conservative | Sancity, Loyalty |
| Lots of people about in Thame. Good ‘Conversations in the street’ about the Food Festival and what I made of Brexit | Sanctity (4.76) | Conservative | None |

**Table 2: Spearman’s Rho correlations between moral foundations**

|  |  | **care** | **fairness** | **loyalty** | **authority** | **sanctity** |
| --- | --- | --- | --- | --- | --- | --- |
| **care** | Correlation coefficient | 1 | .029* | .015* | .017* | .046* |
|  | Sig. (2-tailed) | . | .000 | .010 | .004 | .000 |
| **fairness** | Correlation coefficient | .029* | 1 | .010 | .042* | .019* |
|  | Sig. (2-tailed) | .000 | . | .070 | .000 | .001 |
| **loyalty** | Correlation coefficient | .015* | .010 | 1 | .007 | -.006 |
|  | Sig. (2-tailed) | .010 | .070 | . | .234 | .267 |
| **authority** | Correlation coefficient | .017* | .042* | .007 | 1 | .008 |
|  | Sig. (2-tailed) | .004 | .000 | .234 | . | .154 |
| **sanctity** | Correlation coefficient | .046* | .019* | -.006 | .008 | 1 |
|  | Sig. (2-tailed) | .000 | .001 | .267 | .154 | . |

**Table 3: Log likelihood and significance values per foundation, for the top 10 most frequent words unique to each foundation**

| **Foundation** | **Word freq in foundation** | **Word freq in rest of tweets** | **Log-likelihood** | **χ2**  **Sig.** | | |
| --- | --- | --- | --- | --- | --- | --- |
| **Care** | | | | | | |
| stopbrexit | 460 | 1126 | 72.60 | 0.000 | *** | - |
| help | 411 | 408 | 40.12 | 0.000 | *** | + |
| jobs | 367 | 458 | 9.37 | 0.002 | ** | + |
| economy | 335 | 459 | 2.87 | 0.090 |  | + |
| fight | 330 | 326 | 32.85 | 0.000 | *** | + |
| damage | 311 | 307 | 31.05 | 0.000 | *** | + |
| nhs | 295 | 429 | 0.68 | 0.409 |  | + |
| stop | 275 | 809 | 92.87 | 0.000 | *** | - |
| libdems | 262 | 576 | 23.05 | 0.000 | *** | - |
| hard | 254 | 781 | 100.05 | 0.000 | *** | - |
| **Fairness** | | | | | | |
| law | 284 | 0 | 1046.75 | 0.000 | *** | + |
| communities | 175 | 0 | 640.99 | 0.000 | *** | + |
| offer | 148 | 97 | 255.92 | 0.000 | *** | + |
| leave | 141 | 1164 | 23.93 | 0.000 | *** | - |
| laws | 130 | 0 | 473.67 | 0.000 | *** | + |
| blame | 119 | 5 | 403.81 | 0.000 | *** | + |
| community | 115 | 0 | 417.95 | 0.000 | *** | + |
| fair | 103 | 0 | 373.39 | 0.000 | *** | + |
| honest | 102 | 8 | 325.95 | 0.000 | *** | + |
| trade | 92 | 921 | 36.28 | 0.000 | *** | - |
| **Loyalty** | | | | | | |
| union | 896 | 0 | 571.54 | 0.000 | *** | + |
| customs | 584 | 113 | 52.26 | 0.000 | *** | + |
| getbrexitdone | 559 | 754 | 502.11 | 0.000 | *** | - |
| local | 494 | 20 | 204.39 | 0.000 | *** | + |
| referendum | 479 | 485 | 216.67 | 0.000 | *** | - |
| together | 460 | 4 | 264.36 | 0.000 | *** | + |
| future | 440 | 506 | 274.46 | 0.000 | *** | - |
| great | 426 | 715 | 598.97 | 0.000 | *** | - |
| libdems | 410 | 433 | 206.98 | 0.000 | *** | - |
| good | 409 | 841 | 837.47 | 0.000 | *** | - |
| **Authority** | | | | | | |
| finalsay | 957 | 0 | 278.93 | 0.000 | *** | + |
| tories | 917 | 0 | 266.86 | 0.000 | *** | + |
| minister | 791 | 211 | 35.72 | 0.000 | *** | - |
| theresa | 1429 | 0 | 421.52 | 0.000 | *** | + |
| prime | 776 | 7 | 183.20 | 0.000 | *** | + |
| boris | 618 | 0 | 176.81 | 0.000 | *** | + |
| public | 550 | 561 | 825.61 | 0.000 | *** | - |
| right | 546 | 570 | 853.14 | 0.000 | *** | - |
| voted | 533 | 773 | 1425.20 | 0.000 | *** | - |
| stop | 508 | 580 | 923.85 | 0.000 | *** | - |
| **Sanctity** | | | | | | |
| food | 257 | 0 | 1106.731656 | 0.000 | *** | + |
| security | 201 | 0 | 863.2640401 | 0.000 | *** | + |
| immigration | 174 | 0 | 745.9291255 | 0.000 | *** | + |
| trade | 99 | 894 | 1.882170886 | 0.170 |  | - |
| clean | 83 | 0 | 350.971415 | 0.000 | *** | + |
| leave | 82 | 1125 | 28.32229276 | 0.000 | *** | - |
| bill | 70 | 2 | 287.1503469 | 0.000 | *** | + |
| future | 67 | 872 | 18.60686036 | 0.000 | *** | - |
| being | 65 | 758 | 10.62976194 | 0.001 | ** | - |
| money | 64 | 238 | 24.05826085 | 0.000 | *** | + |
